# The Aryl Hydrocarbon Receptor Undergoes Chaperone-Mediated Autophagy in Triple-Negative Breast Cancer Cells

**DOI:** 10.3390/ijms22041654

**Published:** 2021-02-06

**Authors:** Jinyun Chen, Yujie Yang, Wade A. Russu, William K. Chan

**Affiliations:** Department of Pharmaceutics & Medicinal Chemistry, Thomas J. Long School of Pharmacy, University of the Pacific, Stockton, CA 95211, USA; jinyunc@vet.upenn.edu (J.C.); y_yang16@u.pacific.edu (Y.Y.); wrussu@pacific.edu (W.A.R.)

**Keywords:** aryl hydrocarbon receptor, chaperone-mediated autophagy, LAMP2A, protein degradation, CMA motif

## Abstract

The aryl hydrocarbon receptor (AHR) is a ligand-activated signaling molecule expressed in many cell types, including triple-negative and non-triple-negative breast cancer cells. It affects breast cancer growth and crosstalk with estrogen receptor signaling. Normally, this receptor is degraded shortly after ligand activation via the 26S proteasome. Here, we report that AHR undergoes chaperone-mediated autophagy in MDA-MB-468 triple-negative breast cancer cells. This lysosomal degradation of AHR exhibits the following characteristics: (1) it is triggered by 6 amino-nicotinamide, starvation, and piperazinylpyrimidine compound Q18; (2) it is not observed in non-triple-negative breast cancer cells (MCF-7, T47D, and MDA-MB-361); (3) it can be inhibited by progesterone receptor B but not estrogen receptor alpha; (4) it can be reversed by chloroquine but not MG132; (5) it requires LAMP2A; and (6) it involves AHR-HSC70 and AHR-LAMP2A interactions. The NEKFF sequence localized at amino acid 558 of human AHR appears to be a KFERQ-like motif of chaperone-mediated autophagy, responsible for the LAMP2A-mediated AHR protein degradation.

## 1. Introduction

Autophagy plays an important role in various normal physiological functions, such as immune responses, liver and cardiac function, aging, and responses to infection [1]. In addition, it is implicated in disease development such as cancer, metabolic disorders, and neurodegeneration. Autophagy can be categorized into macroautophagy, microautophagy, and chaperone-mediated autophagy [2]. Macroautophagy generally involves the sequestration of organelles into the formation of the double-membrane structure of autophagosome, followed by lysosomal fusion for degradation. Microautophagy involves the lysosomal internalization of the whole organelle, such as mitochondria and peroxisomes, and a region of cytosol for degradation. Chaperone-mediated autophagy (CMA) allows the selective degradation of client proteins by guiding them directly into lysosomes for degradation (for general review, see [3]). This CMA-mediated lysosomal entry requires the HSC70 chaperone to bring client proteins to the CMA receptor LAMP2A at the lysosomal membrane [4]. This client protein-LAMP2A interaction starts the process of unfolding the client protein before internalization into lysosomes for degradation by hydrolytic enzymes [5]. CMA client proteins contain the HSC70 interaction motif, which has been mapped as a pentapeptide sequence initially shown as KFERQ [6]. Since then, many KFERQ-like motifs have been reported which follow the general rules of this CMA signature motif.

The aryl hydrocarbon receptor (AHR) is a ligand-activated transcription factor essential for a variety of cellular functions; for example, this receptor is involved in xenobiotic sensing [7], normal physiological functions (such as liver development [8,9], immune response [10,11], and hematopoietic stem cell differentiation [12]), and many diseases (such as breast and other cancers [13,14,15], cardiac disorders [16,17], respiratory disorders [18,19], metabolic disorders [20,21], and macular degeneration [22,23]). AHR resides in the cytoplasm as a complex containing AHR, a dimer of HSP90, p23, and XAP2 [24,25,26]. The canonical signaling mechanism of this receptor is initiated by ligand binding to AHR, causing the conformational change necessary for revealing the nuclear localization sequence of AHR. The nuclear internalization of the AHR complex can then occur via a pendulin-dependent mechanism [27]. The binding of aryl hydrocarbon receptor nuclear translocator (ARNT) to AHR dissociates the AHR complex [28], resulting in an active transcription factor in the form of AHR/ARNT heterodimer. This transcription factor then binds to the dioxin response element (DRE) enhancer, activating the transcription of a battery of target gene—most notably is the cytochrome P450 genes *cyp1a1*, *cyp1a2*, and *cyp1b1* [29]. Interestingly, another heterodimeric complex of AHR—AHR/RelB—has been reported to regulate different AHR-dependent gene transcription [30].

After ligand activation, AHR undergoes ubiquitination and is degraded via the 26S proteasome as early as 1–2 h after ligand treatment [31]. The displacement of HSP90 from AHR in the cytoplasm by geldanamycin (GA) also causes the degradation of AHR via the same ubiquitin-proteasome system [32]. We observed that the down-regulation of p23 promotes AHR protein degradation without ligand treatment [33], unveiling the potential dynamics of AHR protein synthesis and degradation in maintaining the AHR cellular levels. However, the mechanisms responsible for the degradation of AHR without the addition of an exogenous ligand are largely uncharacterized.

**Figure 1:**
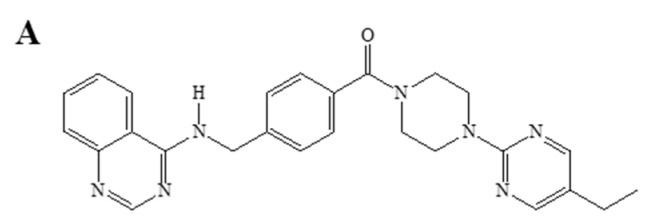

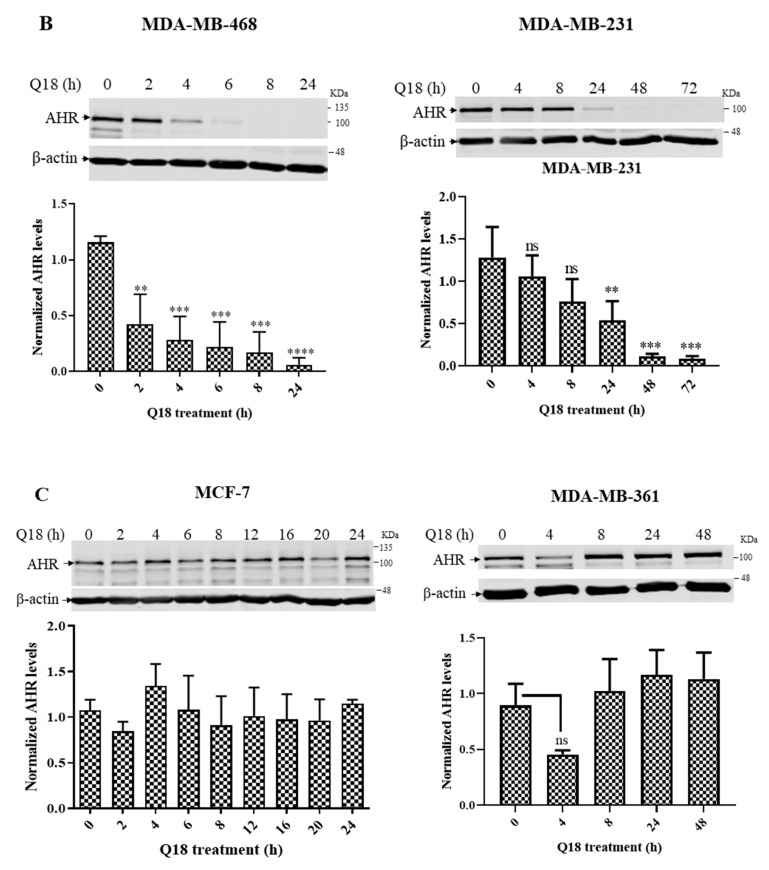
Western blot analysis of the AHR protein levels in TNBC and non-TNBC cells after Q18 treatment. (**A**) chemical structure of Q18. (**B**) MDA-MB-468 and MDA-MB-231 (TNBC). (**C**) MCF-7 and MDA-MB-361 (non-TNBC). Cells were treated with 10 μM of Q18 or DMSO (vehicle) for the indicated times. All lanes contain 60 μg of whole cell lysates. The amount of AHR was normalized by β-actin. The normalized value of AHR from Q18 treatment was divided by the normalized value from DMSO treatment to calculate the relative abundance of AHR at each timepoint. Y-axis represents the fold difference in Q18 over DMSO at each timepoint. Results are means ± SD of three independent experiments. One-way ANOVA with Dunnett’s multiple comparisons test was used to determine statistical significance. ** *p* < 0.01, *** *p* < 0.001, **** *p* < 0.0001 when compared with zero timepoint in MDA-MB-468 and MDA-MB-231 cells. ns, not significant. MCF-7 and MDA-MB-361 data were all statistically insignificant when compared with the zero timepoint.

Q18 (with the structure shown in Figure 1A) is a synthetic piperazinylpyrimidine molecule which was originally designed as a tyrosine kinase inhibitor [34]. Although there is no tyrosine kinase target identified thus far, Q18 appears to alter the AHR function. With the use of Q18, we serendipitously unveiled a new mechanism that degrades the AHR protein. Here, we provide evidence supporting that AHR is a CMA client protein which undergoes lysosomal degradation in triple-negative breast cancer (TNBC) cells.

## 2. Results

### 2.1. Suppression of the AHR Protein Levels in Triple-Negative, but Not in Non-Triple-Negative, Breast Cancer Cells by Q18

We treated two TNBC and two non-TNBC cells with 10 µM of Q18 for various time periods to determine its effect on the AHR protein levels. We observed that the AHR protein levels were significantly suppressed after Q18 treatment by 50% as early as 2 and 24 h, respectively, in triple-negative MDA-MB-468 and MDA-MB-231 cells (Figure 1B). This suppression was not observed in non-triple-negative MCF-7 cells within a 24 h treatment period (Figure 1C, left). Although we observed a 50% suppression of the AHR protein levels by Q18 in non-triple-negative MDA-MB-361 cells, the effect was transient—the AHR levels were reverted back to normal in 4 h (Figure 1C, right). Collectively, the AHR protein levels were uniquely suppressed by Q18 in TNBC cell lines.

### 2.2. Degradation of AHR in Triple-Negative Breast Cancer Cells Is Dependent on Progesterone Receptor but Not Estrogen Receptor

**Figure 2:**
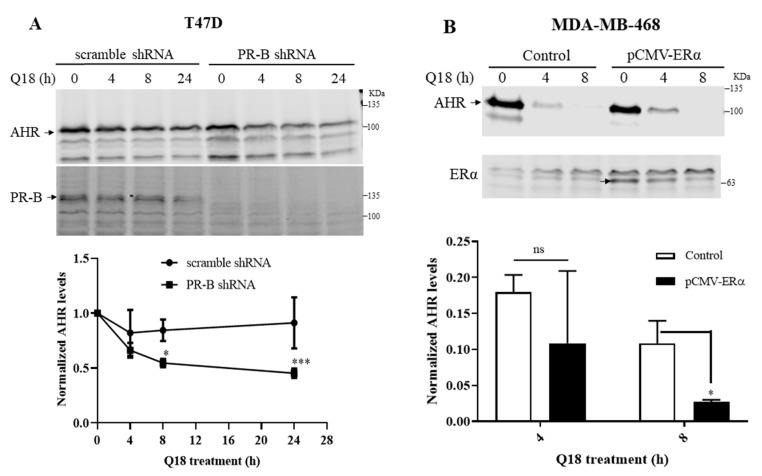

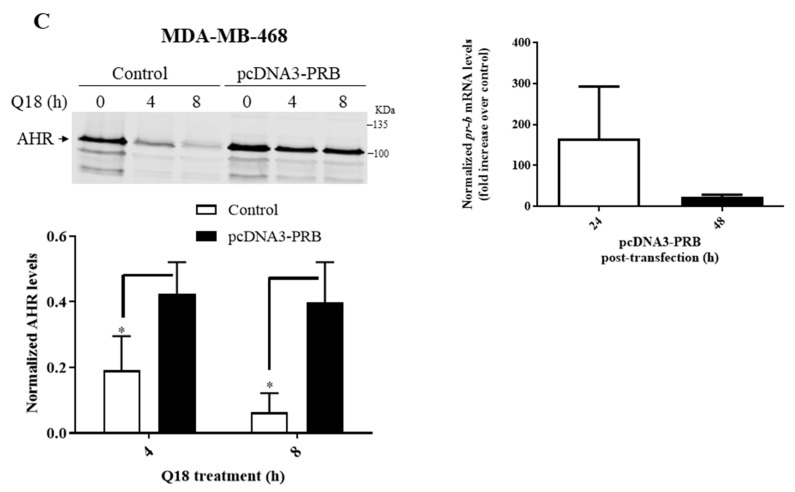
Effect of PR-B and ERα on the Q18-mediated suppression of the AHR protein levels. Timecourse Q18 treatment of: (**A**) T47D stable cells carrying either PR-B-specific shRNA or scramble shRNA. AHR at zero time was arbitrarily set to one for comparison. (**B**) MDA-MB-468 cells transiently transfected with the plasmid carrying the full length ERα cDNA (pCMV-ERα). Control represents non-transfected cells. (**C**, left panel) MDA-MB-468 cells transiently transfected with the plasmid carrying the full length PR-B cDNA (pcDNA3-PR-B) or the pcDNA3 plasmid which was derived from pcDNA-PR-B after Srf I and Eco RV digest to remove 75% of PR-B cDNA (control). All lanes for Western blot analysis contain 60 μg of whole cell lysates. All Western blot (**A**–**C**) data of AHR were normalized by total protein stain. All the results are means ± SD of three independent experiments. Two-way ANOVA with Sidak’s multiple comparisons test was used in 2A for statistical analysis. * *p* < 0.05, *** *p* < 0.001 when compared between PR-B shRNA and scramble shRNA. Unpaired *t* test was used in 2B and 2C for statistical analysis. ns, not significant. * *p* < 0.05 when compared to control. The *pr-b* message levels were determined by RT-qPCR 24 or 48 h post-transfection, normalized by 18S (**C**, right panel). Cq of *pr-b* message 24 h after post-transfection were 32–33 whereas 48 h after post-transfection were 35–36. The Cq of *pr-b* message in untransfected cells were all above 39. Y-axis represents a fold increase in *pr-b* messages of PR-B transfected over empty plasmid transfected. Although the PR-B-transfected cells consistent showed more *pr-b* message, all Cq numbers were beyond 30, showing that the amount of all *pr-b* messages were all very low so that no statistics was performed.

Since TNBC cells do not express estrogen receptor α (ERα) and progesterone receptor B (PR-B), we performed transient transfection experiments to alter the cellular ERα or PR-B levels in an effort to address whether the Q18 suppression of the AHR protein levels is ERα or PR-B dependent. We observed that the down-regulation of the PR-B levels in PR-B positive T47D cells using the PR-B-specific short hairpin RNA (shRNA) sensitized the Q18 suppression of the AHR protein levels (Figure 2A), supporting the notion that the absence of PR-B in MDA-MB-468 cells allows the downregulation of AHR by Q18 to occur. Conversely, the exogenous expression of ERα in MDA-MB-468 cells had no effect on the Q18-mediated AHR protein degradation (Figure 2B), ruling out the involvement of ERα in this Q18 effect. Although it was very evident that Q18 failed to alter the AHR protein levels in MDA-MB-468 cells after the transient transfection of a PR-B-expressing plasmid (Figure 2C, left), we were unable to detect any exogenous PR-B expression by Western blot analysis after transfection. In this experiment, we compared the AHR levels of cells transfected with a PR-B-expressing plasmid with cells transfected with the same plasmid, except that 75% of the PR-B cDNA was removed (which we referred to as the control). The transfected *pr-b* message levels were at a very low level, as reflected by a Cq number beyond 30 (Figure 2C, right). Nevertheless, the endogenous *pr-b* message levels were consistently two orders of magnitude lower than the transfected levels at 24 h after transfection. It is conceivable that very small amounts of PR-B expression in MDA-MB-468 cells would be sufficient to suppress the Q-18-mediated AHR degradation.

### 2.3. Q18 is an AHR Antagonist

**Figure 3:**
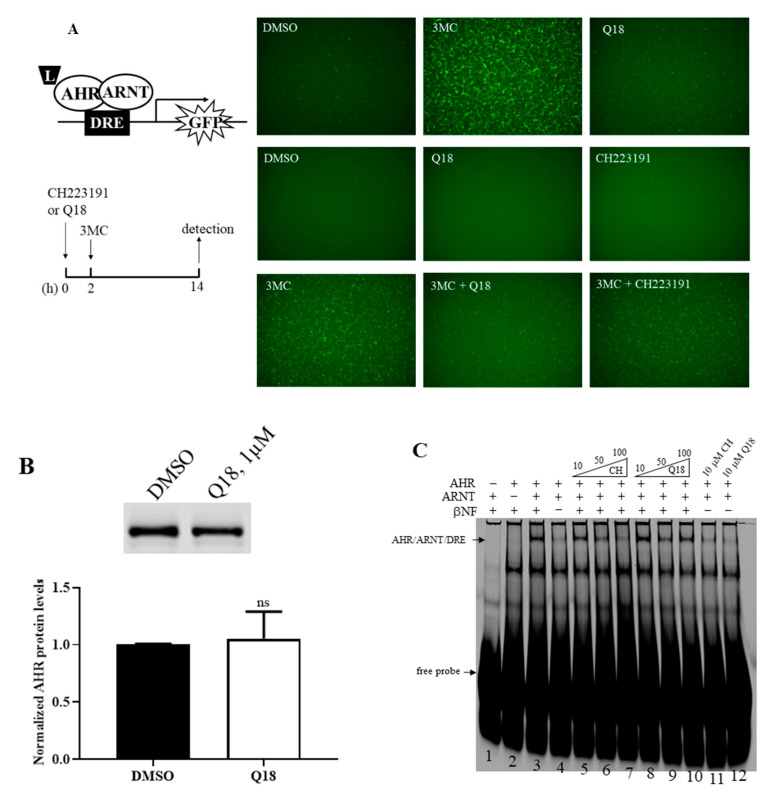
Effect of Q18 on AHR ligand binding. (**A**) Treatment of Q18, CH223191, and 3MC in rat H4G1.1c3 cells stably transfected with a plasmid carrying a DRE-driven GFP cDNA (left top diagram). Cells were treated for 12–14 h (as indicated in the left bottom timeline diagram) with DMSO, 1 μM 3MC, 1 μM Q18, 10 μM CH223191, 1 μM 3MC plus 1 μM Q18, or 1 μM 3MC plus 10 μM CH223191. Q18 could not cause GFP formation but could block the GFP formation caused by 3MC. CH223191 was used as the AHR antagonist control. Top panel represents one experiment; middle and bottom panels represent another experiment. These two experiments were repeated two more times with similar results. (**B**) Western blot analysis to show the effect of Q18 on the AHR protein levels when the stable H4G1.1c3 cells were treated with 1 μM of Q18 for 24 h. The AHR protein levels were normalized by total protein stain. The error bar represents the mean ± SD of three independent experiments and the representative Western images are shown. (**C**) Effect of Q18 on the formation of the AHR/ARNT/DRE gel shift complex. Gel shift complex was formed in the presence of 10 μM of βNF as the AHR ligand (lanes 1–4). A total of 10 μM of Q18 could not form the AHR gel shift complex but could suppress the βNF-dependent AHR gel shift formation at a 50 μM concentration (lanes 8–10 and 12). CH223191 (CH) was used as the AHR antagonist control (lanes 5–7 and 11). Arrows indicate the AHR/ARNT/DRE gel shift complex and free DRE conjugated with IRD700 (free probe). This gel shift experiment was repeated once with similar results. ns, not significant.

Next, we addressed whether Q18 could act as an AHR ligand and, in turn, cause AHR protein degradation. We treated the rat H4G1.1c3 cells stably transfected with a plasmid carrying a DRE-driven GFP cDNA with 1 μM of Q18—a 10-fold lower concentration than what was used for the MDA-MB-468 cell study, since 10 μM of Q18 caused significant H4G1.1c3 cell death. We detected minimal GFP expression when comparing with the treatment of an AHR ligand 3MC at a 1 μM concentration for 12 h, suggesting that Q18 is not an AHR ligand and cannot activate the DRE-driven gene transcription (Figure 3A, top panel). When we pre-treated the cells with 1 μM of Q18 two hours before the 1 μM 3MC treatment, we observed that there was noticeable reduction in GFP fluorescence in the presence of Q18, suggesting that Q18 acts as an AHR antagonist, analogous to the AHR antagonist CH223191 (Figure 3A, middle and bottom panels). Q18 did not alter the AHR protein levels in H4G1.1c3 cells (Figure 3B), suggesting that the lack of GFP expression in the presence of Q18 in H4G1.1c3 cells is not due to a lesser amount of AHR to activate the GFP gene transcription. Next, we examined whether Q18 could cause the ligand-dependent AHR gel shift formation. We used the Pichia-expressed human AHR and ARNT [35] to form the AHR gel shift complex, which was βNF-, AHR-, and ARNT-dependent (Figure 3C, lanes 1–4). Co-chaperone p23 was used to promote the gel shift formation [36]. We observed that, unlike an AHR ligand βNF, Q18 was not able to generate the AHR gel shift complex at a 10 µM concentration (Figure 3C, lane 12), consistent with our rat H4G1.1c3 cell study that showed that Q18 is not an AHR ligand. Q18 could, however, inhibit the formation of the βNF-dependent AHR gel shift complex at a 50 μM concentration, although higher concentrations did not show more inhibition (Figure 3C, lanes 8–10). The AHR antagonist CH223191 was used as the control to show the concentration-dependent inhibition of the βNF-dependent AHR gel shift complex formation (Figure 3C, lanes 5–7). Collectively, data from the DRE-driven GFP expression and gel shift studies supported that Q18 is an AHR antagonist which competes with an AHR ligand in binding to the ligand-binding domain of AHR.

### 2.4. Q18 Promotes the Autophagy-Mediated AHR Protein Degradation and Does Not Affect the AHR Transcript Levels

**Figure 4:**
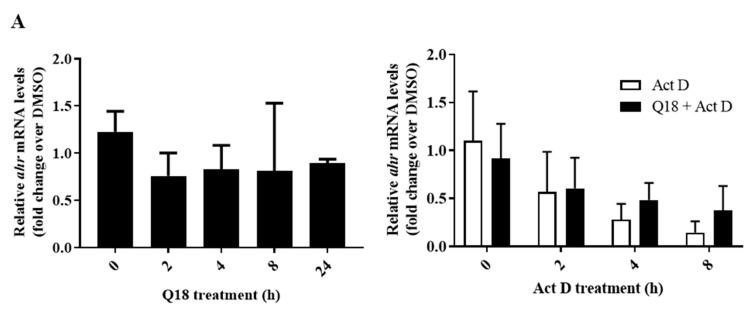

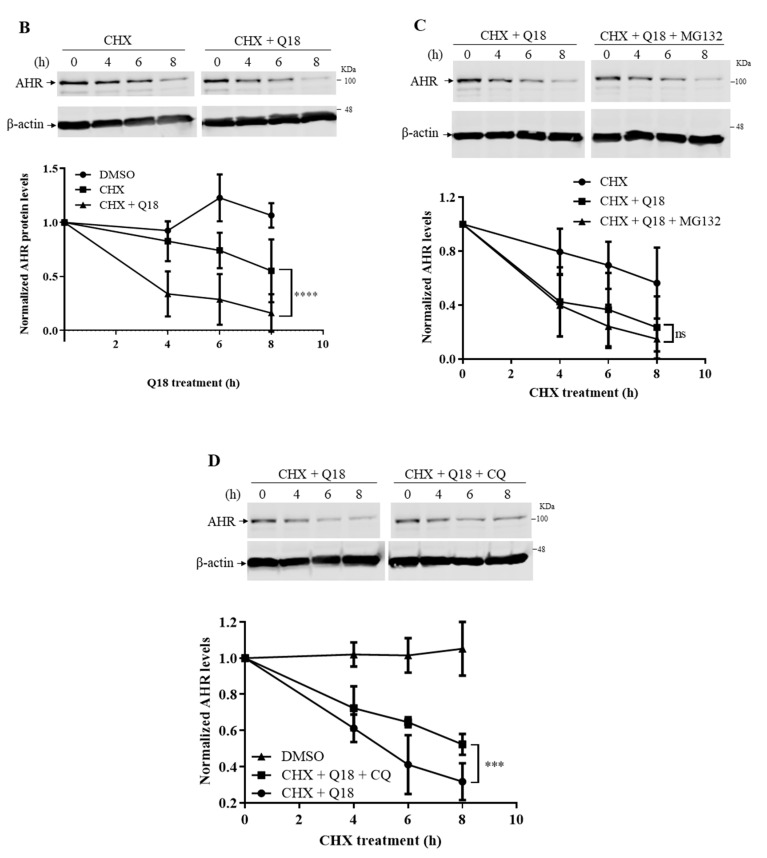
Effect of Q18 on AHR gene transcription, message stability, and protein stability in MDA-MB-468 cells. (**A**) The *ahr* message levels were measured after 10 μM of Q18 or DMSO (vehicle) treatment for 0–24 h (left). The half-life of the *ahr* message was determined in the presence of 5 μg/mL of actinomycin D (Act D) or DMSO (vehicle) with or without 10 μM of Q18 treatment (right). The relative *ahr* mRNA levels were determined using 18S and DMSO as vehicle control (2^−ΔΔ*C*q^ method). One-way ANOVA with Dunnett’s multiple comparisons test was used in analyzing the Q18 treatment data (left). Unpaired *t* test was used in analyzing the Act D +/− Q18 treatment data (right). Results are means ± SD of: (left) *n* = 6 for zero, 4 h, and 8 h timepoints; *n* = 3 for 2 h and 24 h timepoints; (right) *n* = 3. *n* represents the number of independent experiments. All the data were statistically insignificant. (**B**) The AHR protein half-life was determined in the presence of 30 μg/mL of cycloheximide (CHX) with or without 10 μM of Q18 treatment. The images are representative of the data. Two-way ANOVA with Tukey’s multiple comparisons test was used for the statistical analysis. **** *p* < 0.0001 showed that there is a significant difference in the AHR protein levels in CHX-treated cells versus CHX plus Q18-treated cells. ns, not significant. (**C**) same as (**B**) +/− 10 μM of MG132. Results are means ± SD of seven independent experiments. Two-way ANOVA with Turkey’s multiple comparisons test was used for statistical analysis. There is no difference in the AHR degradation in the CHX plus Q18 group vs. CHX plus Q18 plus MG132 group (*p* = 0.4582). (**D**) same as (**B**) +/− 20 μM of chloroquine (CQ). Results are means ± SD of: *n* = 3 for DMSO group; *n* = 4 for CHX plus Q18 and CHX plus Q18 plus CQ group. *n* represents the number of independent experiments. Two-way ANOVA with Tukey’s multiple comparisons test was used in statistical analysis. *** *p* < 0.001 compared between CHX plus Q18 and CHX plus Q18 plus CQ-treated cells.

Next, we investigated whether the suppression of the AHR protein levels by Q18 is caused by a reduction in the AHR transcript and/or the promotion of the AHR protein degradation. We observed that there was no statistically significant difference in the AHR transcript levels in the presence of 10 µM of Q18 in MDA-MB-468 cells when comparing each timepoint (2–24 h) with the zero timepoint (Figure 4A, left). We performed an actinomycin D experiment to determine whether Q18 could increase the degradation of the AHR transcript in MDA-MB-468 cells. We observed that the AHR transcript was degraded similarly in the presence or absence of Q18 when transcription was inhibited by actinomycin D (Figure 4A, right). Collectively, our data suggested that the suppression of the AHR protein levels by Q18 is not mediated through transcriptional and RNA stability mechanisms. Next, we measured the AHR protein half-life in MDA-MB-468 cells when protein synthesis was inhibited by 30 µg/mL of cycloheximide. We observed that AHR protein was degraded significantly faster in the presence of Q18 (Figure 4B), supporting the notion that Q18 triggers AHR protein degradation. This Q18-dependent AHR protein degradation was not affected by the proteasome inhibitor MG132, since the presence of MG132 did not significantly change the AHR protein half-life (Figure 4C), suggesting that proteasomal degradation is not involved in the Q18-mediated AHR degradation. On the contrary, the degradation of the AHR protein by Q18 was retarded significantly in the presence of the autophagy inhibitor chloroquine (Figure 4D), showing that Q18 causes the lysosomal degradation of AHR.

### 2.5. Degradation of AHR in Triple-Negative Breast Cancer Cells Is LAMP2A-Mediated

**Figure 5:**
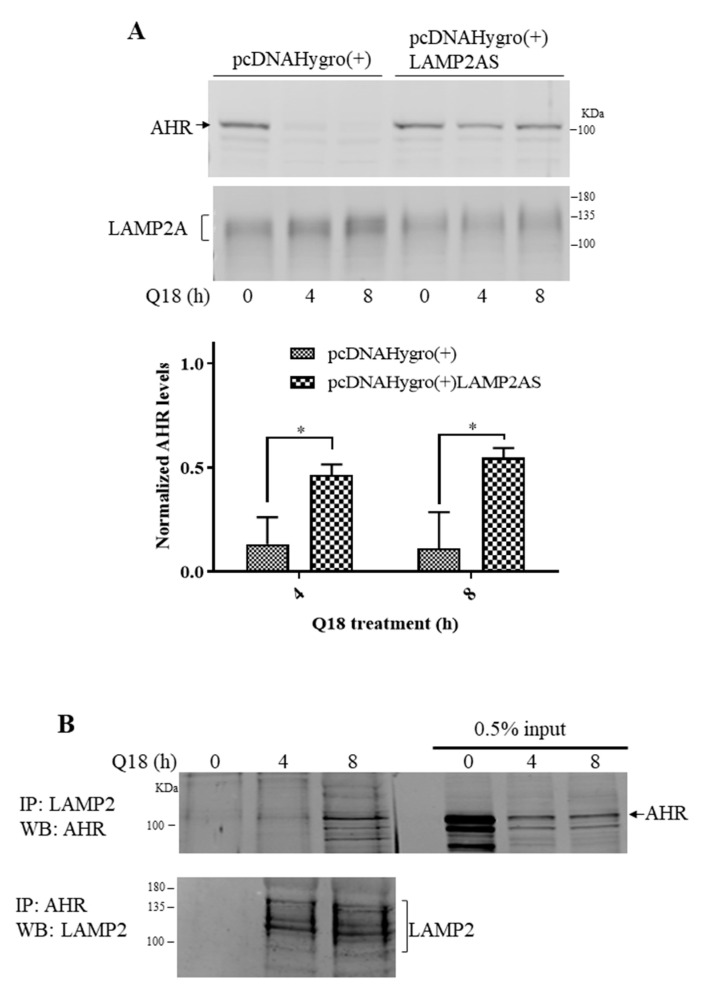

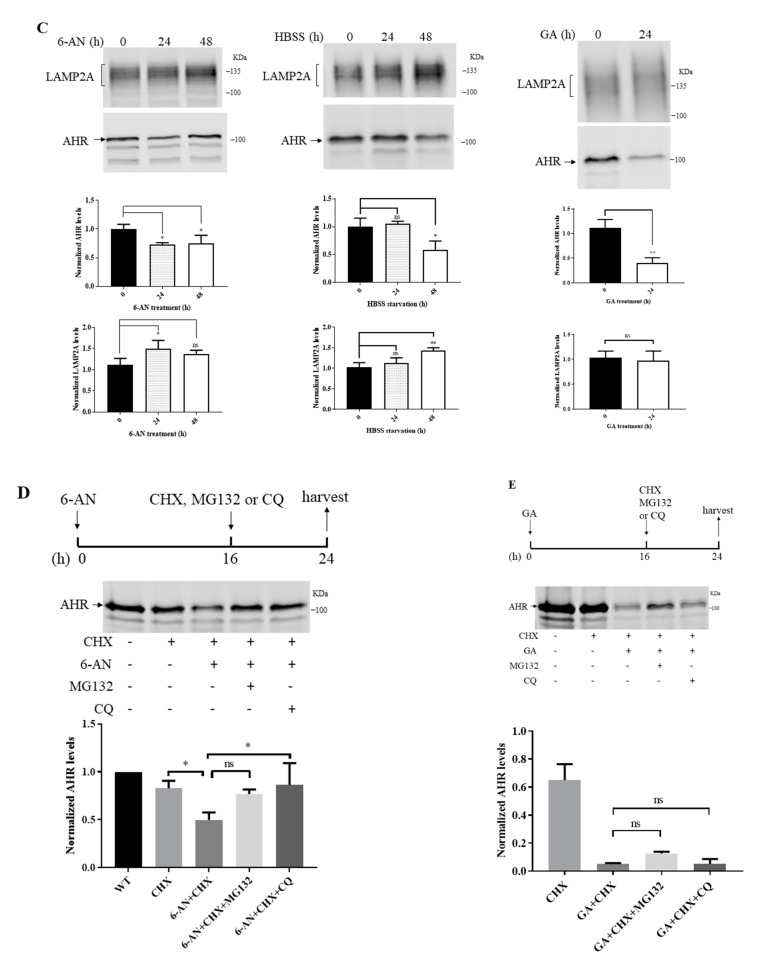

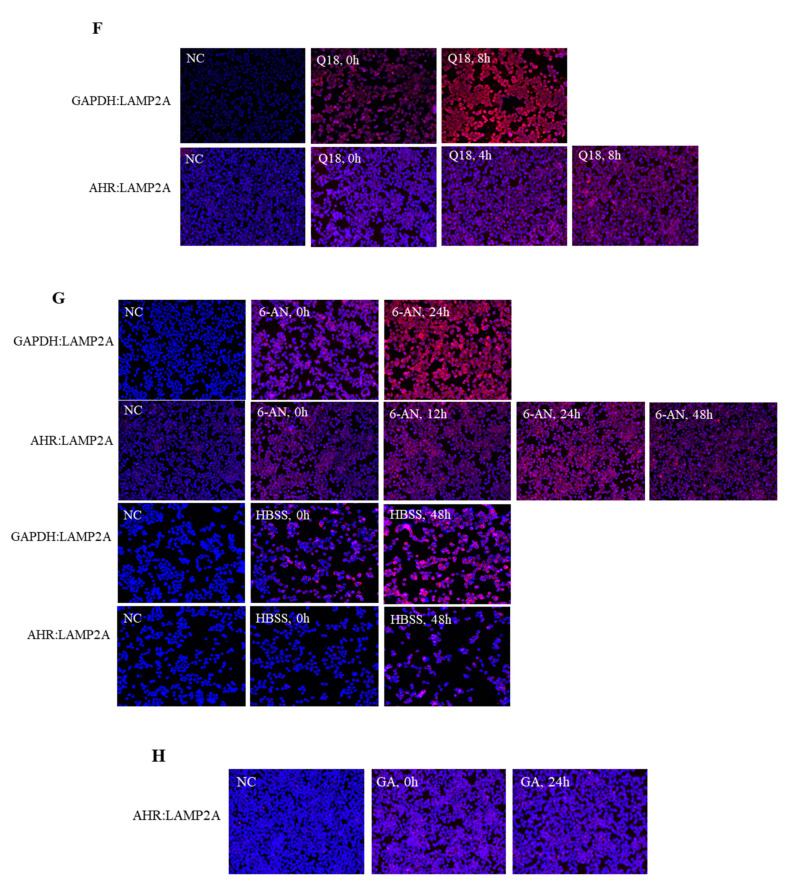

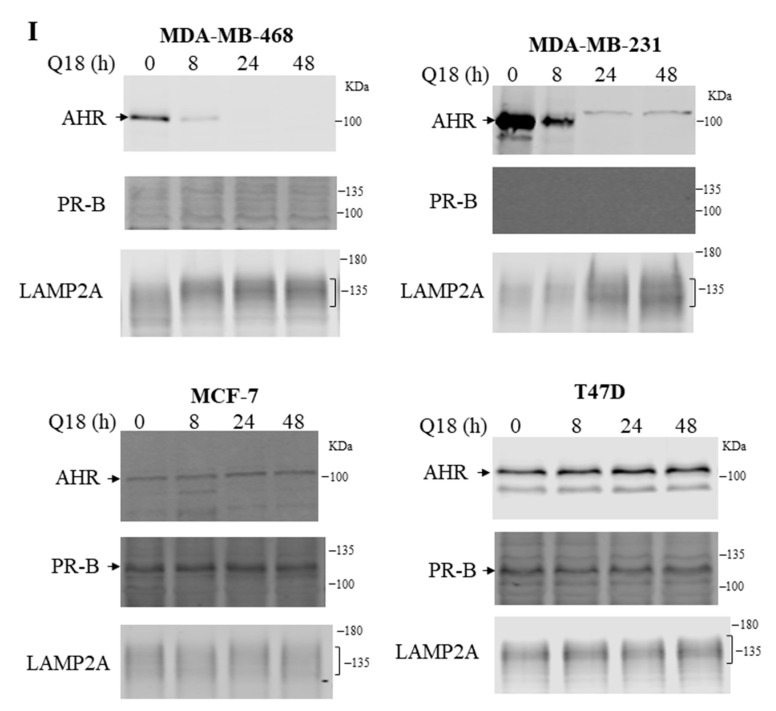
Effect of the LAMP2-mediated CMA on AHR degradation. (**A**) MDA-MB-468 cells were transiently transfected with either empty plasmid pcDNAHydro(+) or pcDNAHydro(+) LAMP2AS carrying the LAMP2-specific antisense and treated with 10 μM of Q18 for 0–8 h. Zero timepoints were arbitrarily set as 1 to normalize the AHR amount remaining at 4 and 8 h. Y-axis represents the amount of AHR remaining. Results are means ± SD of three independent experiments. Unpaired *t* test was used for statistical analysis. * p < 0.05 when comparing the AHR levels of LAMP2A knockdown with empty plasmid transfected cells. The amount of AHR was normalized by total protein stain, whereas each lane contained 60 μg of protein loaded. (**B**) MDA-MB-468 cells were treated with 10 μM of Q18 for 0, 4, or 8 h, followed by the immunoprecipitation of 2 mg of whole cell lysates with either AHR or LAMP2 antibody. IP represents the antibody used to perform the immunoprecipitation, whereas WB represents the Western blot analysis of protein shown. Input represents 0.5% of sample to start the experiment. (**C**) 10 μM of 6-AN, HBSS (mimic starvation), or 1 μg/mL of GA were added to MDA-MB-468 cells for 0, 24, or 48 h (0 or 24 h for GA treatment). Results are means ± SD of three independent experiments. The amount of AHR and LAMP2A was normalized by total protein stain, whereas each lane contained 60 μg of protein loaded. One-way ANOVA with Dunnet’s multiple comparisons test (6-AN and HBSS treatment) and unpaired *t* test (GA treatment) were used for statistical analysis. * p < 0.05, ** p < 0.01 when compared to zero timepoint. (**D**) MDA-MB-468 cells was treated with 10 μM of 6-AN for 24 h. ns, not significant. In the last 8 h before harvest, CHX (30 μg/mL), MG132 (10 μM), or CQ (20 μM) were added to the cells (right, top left with timeline diagram). Results are means ± SD of three independent experiments. One-way ANOVA with Tukey’s multiple comparisons test was used for statistical analysis. * p < 0.05 when compared CHX group with 6-AN + CHX group and 6-AN + CHX group with CHX + 6-AN + CQ group. Difference between 6-AN + CHX group and 6-AN + CHX + MG132 group is not significant. (**E**) MDA-MB-468 cells were treated with 1 μg/mL of GA for 24 h (see timeline diagram, top right). CHX (30 μg/mL), MG132 (10 μM), or CQ (20 μM) were added to cells in the last 8 h. Results are means ± SD of three independent experiments. One-way ANOVA with Tukey’s multiple comparisons test was used for statistical analysis. The amount of AHR was normalized by total protein stain, whereas each lane contained 60 μg of protein loaded. (**F**–**H**) Interaction between AHR and LAMP2A, GAPDH, and LAMP2A were tested in MDA-MB-468 cells in situ, followed by 10 μM Q18, 10 μM 6-AN, HBSS, or 1 μg/mL of GA treatment for a period of time. Proximity ligation assay signals were shown as red fluorescent spots around the nucleus (blue). Negative control (NC) was performed without antibodies. Each panel represents either GAPDH-LAMP2A interaction (GAPDH:LAMP2A) or AHR-LAMP2A interaction (AHR:LAMP2A). GAPDH was used as the positive control. AHR-LAMP2A interaction was detected in Q18, 6-AN, and HBSS treatments but not in GA treatment. This experiment was repeated twice with similar results. (**I**) MDA-MB-468, MDA-MB-231, MCF-7, and T47D cells were treated with 10 μM of Q18 or DMSO (vehicle) for 0–48 h. Western blot analysis was performed to detect the AHR, LAMP2, and PR-B levels. Each lane contained 60 μg of protein. This experiment was repeated once with similar results.

Since chloroquine blocked the Q18-mediated AHR protein degradation, we investigated whether LAMP2A, an essential protein responsible for importing client protein into lysosomes for CMA [37], is involved in the degradation of AHR. We used a LAMP2 antibody from Santa Cruz Biotechnology (H4B4, sc-18822) which detects LAMP2A in a broad region. This antibody has been used by other researchers to detect LAMP2A expression [38]. We observed that LAMP2A could be down-regulated in MDA-MB-468 cells via the transient transfection of a plasmid carrying the LAMP2A-specific antisense cDNA. When the LAMP2A expression was suppressed in MDA-MB-468 cells, the degradation of the AHR protein was significantly reversed from 11–13% to 46–55% content up to 8 h of Q18 treatment (Figure 5A). During this 8 h Q18 treatment of the empty plasmid transfected cells, the amount of the LAMP2A protein increased while AHR was substantially reduced, suggesting that Q18 may upregulate LAMP2A, leading to AHR degradation. Next, we addressed whether the accumulated LAMP2A could interact with AHR. We performed a co-immunoprecipitation experiment and observed that AHR was co-precipitated with LAMP2A and vice versa in a time-dependent manner when cells were treated with Q18 (Figure 5B). In addition, the activation of CMA by 6-AN or starvation (via HBSS treatment) also upregulated the LAMP2A expression and degraded AHR in MDA-MB-468 cells, although the timing of the AHR suppression varied between these two treatments (Figure 5C). However, a reduction in the AHR levels by GA, a reported CMA activator, did not show any apparent difference in the LAMP2A protein levels. Results from the Western blot analysis showed that CQ could significantly block the 6-AN-dependent AHR degradation in the cycloheximide experiment (Figure 5D). AHR might be partially degraded by 26S proteasome after 6-AN treatment, since MG132 was able to reverse some of the AHR degradation, but this effect was not statistically significant (Figure 5D). On the contrary, the treatment of MDA-MB-468 cells with GA showed a more pronounced degradation of AHR which was not reversed by either CQ or MG132 (Figure 5E).

### 2.6. Treatment of Q18, 6-AN or Starvation in MDA-MB-468 Cells Causes Interaction between AHR and LAMP2A in Proximity Ligation Assay

Next, we examined whether the activation of CMA in MDA-MB-468 cells could induce the AHR-LAMP2A interaction using a proximity ligation assay to detect interaction in situ. GAPDH, which is a CMA client protein [39], was used as a positive control to show the interaction between LAMP2A and client protein during CMA. The treatment of Q18 increased the interaction between GAPDH and LAMP2A, showing that Q18 triggers CMA in MDA-MB-468 cells after 8 h of treatment (Figure 5F). Likewise, the interaction between AHR and LAMP2A was noticeably increased when the cells were treated with Q18 for 8 h. Other means of CMA activation by either 6-AN or starvation also showed a clear increase in the AHR-LAMP2A interaction at 24 and 48 h, respectively (Figure 5G). CMA activation by 6-AN and starvation was confirmed using the GADPH control. However, we failed to detect any interaction between AHR and LAMP2A when MDA-MB-468 cells were treated with 1 μg/mL of GA for 24 h (Figure 5H).

### 2.7. Treatment of Q18 Increases the LAMP2A Expression in TNBC but Not in Non-TNBC Cells

Next, we examined whether the LAMP2A expression could be upregulated by Q18 to cause AHR degradation. We observed that Q18 treatment for up to 48 h increased the LAMP2A expression in TNBC (MDA-MB-468 and MDA-MB231) but not in non-TNBC (MCF-7 and T47D) cells (Figure 5I). PR-B expression was detected in non-TNBC, which was not affected by Q18 treatment. The expression of PR-B was not detected in TNBC cells.

### 2.8. 6-AN Promotes AHR-HSC70 and AHR-LAMP2A Interaction in MDA-MB-468 Cells

**Figure 6:**
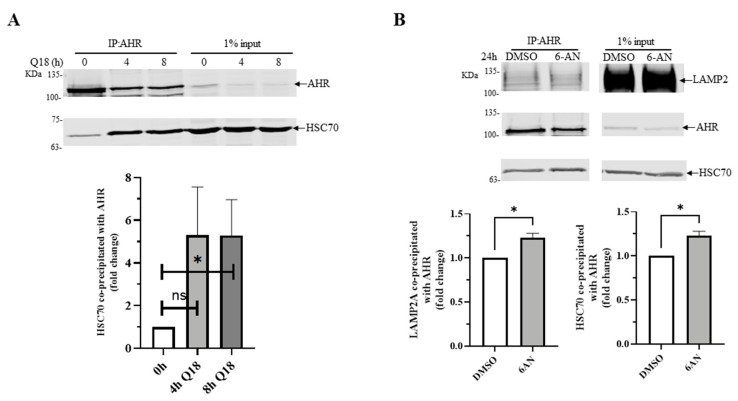
Effect on AHR-HSC70 interaction in the presence or absence of Q18 or 6-AN in MDA-MB-468 cells. Cells were treated with (**A**) 10 μM of Q18 for 0, 4, or 8 h or (**B**) DMSO or 10 μM of 6-AN for 24 h, followed by the immunoprecipitation of 2 mg of whole cell lysates with AHR antibody SA-210 (IP: AHR). The Western bands were detected using protein-specific antibodies (SA-210 for AHR, B-6 for HSC70, and H4B4 for LAMP2A). Input represents 1% of sample to start the experiment. One control (0 h for **A** and DMSO for **B**) of each data set was arbitrarily set as one to normalize the three independent experimental data sets, thus no error bar was presented for these conditions. Results are means ± SD of three independent experiments. Two-tailed unpaired *t* test with Welch’s correction were used for statistical analysis. * p < 0.05 when compared to the control. ns, not significant.

It has been reported that HSC70 escorts cargo to LAMP2A during CMA [3]. Therefore, we examined whether AHR might interact with HSC70 in a manner that is dependent upon CMA activation. We performed co-immunoprecipitation experiments using anti-AHR SA-210 in MDA-MB-468 cells and observed that AHR interacted with HSC70 with or without CMA activation by Q18 or 6-AN. However, either 10 μM of Q18 or 6-AN significantly enhanced the AHR-HSC70 interaction by 5-fold and 1.25-fold, respectively (Figure 6A,B), showing that the activation of CMA can increase the interaction between AHR and HSC70. Similar to the earlier data showing that Q18 triggered the AHR-LAMP2 interaction (Figure 5B), the activation of CMA by 6-AN showed a small (but significant) quantifiable increase in the interaction between AHR and LAMP2 (Figure 6B).

### 2.9. AHR Contains a CMA Signature Motif

**Figure 7:**
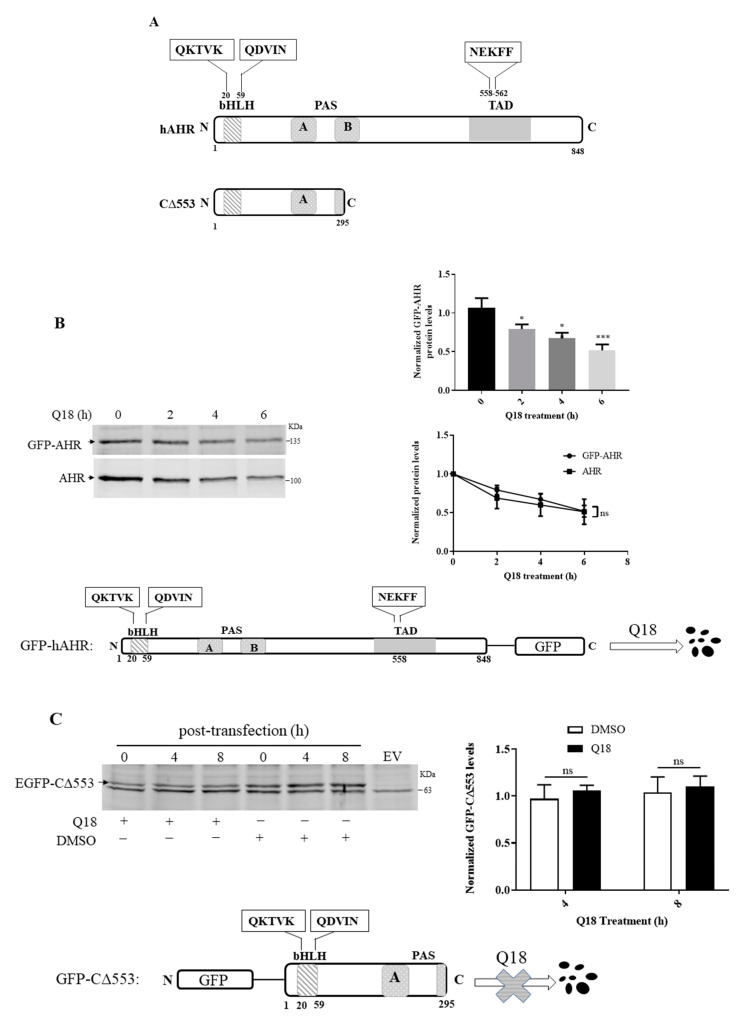

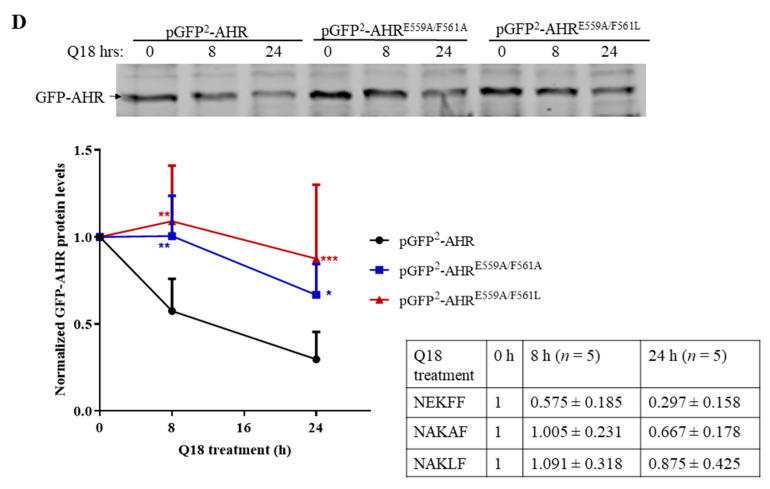
Identification of CMA motif of human AHR. (**A**) Schematic of human AHR (hAHR) and AHR construct CΔ553 indicating the location of the three putative CMA motifs: QKTVK, QDVIN, and NEKFF. (**B**) MDA-MB-468 cells were transiently transfected with pGFP^2^-N2-AHR. Cells were then treated with 10 μM of Q18 for 0–6 h. Results are means ± SD of three independent experiments. One-way ANOVA with Dunnett’s multiple comparisons test (bar graph) and two-way ANOVA with Sidak’s multiple comparisons test (line graph) were used for statistical analysis. * p < 0.05, ** p < 0.01, and *** p < 0.001 when compared with zero timepoint. ns, not significant. There is no difference between GFP-AHR and endogenous AHR degradation. Bottom diagram and top left images show the degradation of the transfected GFP-AHR upon Q18 treatment in MDA-MB-468 cells. The amount of AHR and GFP-AHR was normalized by total protein stain, whereas each lane contained 60 μg of protein loaded. (**C**) MDA-MB-468 cells were transiently transfected with pEGFP-C2-C∆553 or pEGFP-C2 plasmid (empty vector, EV). Cells were treated with 10 μM of Q18 or DMSO (vehicle) for 0–8 h. The graph represents the normalized amount of GFP-CΔ553 at 4 and 8 h after Q18 or DMSO treatment with the amount at zero hour set as one. Results are means ± SD of three independent experiments. Unpaired *t* test was used for statistical analysis. There is no difference between Q18 and vehicle treatment. EGFP-CΔ553 was stable in the presence of Q18 for up to 8 h. (**D**) MDA-MB-468 cells were transiently transfected with pGFP^2^-N2-AHR (NEKFF, black), pGFP^2^-N2-AHR^E559A/F561A^ (NAKAF, blue), or pGFP^2^-N2-AHR^E559A/F561L^ (NAKLF, red) and then treated with 10 μM of Q18 for 0, 8, or 24 h. Both NAKAF and NAKLF mutants were statistically more stable than GFP-AHR with NEKFF in the presence of Q18. Results are means ± SD (with error bars of above half only) of five independent experiments, except that the 24 h transfection of pGFP^2^-N2-AHR (black) and pGFP^2^-N2-AHR^E559A/F561L^ (red) was from six independent experiments. The table represents the mean values ± SD with zero timepoints set as one for comparison. Two-way ANOVA with Turkey’s multiple comparisons test was used for statistical analysis. * p < 0.05, ** p < 0.01, and *** p < 0.005 when compared with pGFP^2^-N2-AHR-transfected cells.

Human AHR contains three putative sequences—QKTVK, QDVIN, and NEKFF—which follow the general requirements of a CMV signature motif (Figure 7A). When we transiently expressed the GFP fusion of the full-length human AHR in MDA-MB-468 cells, we observed that the GFP-AHR fusion was degraded significantly within six hours of the Q18 treatment (Figure 7B), validating that the GFP fusion of AHR has the same fate as the endogenous AHR when treated with Q18. However, when we monitored the levels of the GFP fusion of an AHR construct (amino acid 1-295 of human AHR, C∆553) which contains two out of the three putative signature motifs; we observed that it remained stable up to 8 h of Q18 treatment in MDA-MB-468 cells (Figure 7C), suggesting that the two putative motifs close to the bHLH domain are not used to trigger AHR degradation. Next, we generated GFP-AHR mutants E559A/F561A and E559A/F561L, which changed the NEKFF motif into NAKAF and NAKLF, respectively. Our transient transfection results revealed that both NAKAF and NAKLF mutants were more resistant to degradation up to 24 h when compared to the NEKFF-containing GFP-AHR in a statistically significant manner, supporting that NEKFF is a KFERQ-like motif that is responsible for AHR undergoing CMA (Figure 7D).

## 3. Discussion

**Figure 8:**
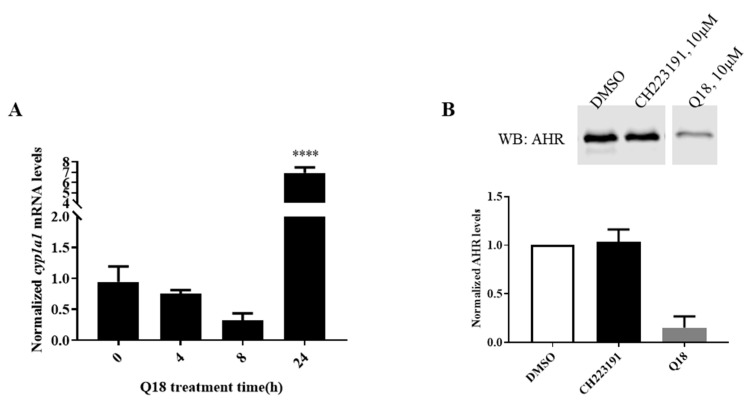
Q18 effect on the cyp1a1 transcript and AHR protein levels in MDA-MB-468 cells. (**A**) Effect on the *cyp1a1* transcript levels when MDA-MB-468 cells were treated with Q18. At each timepoint, cells were treated with either 10 μM of Q18 or with vehicle (DMSO). RT-qPCR was performed as described in the Materials and Methods section. The fold changes of gene transcript levels between Q18 treated and DMSO vehicle control treated cells were normalized by 18S. One-way ANOVA with Dunnett’s multiple comparisons test was used in analyzing the Q18 treatment data. **** *p* < 0.0001 showed that there is a significant difference in the *cyp1a1* mRNA levels at the 24 h timepoint versus zero timepoint. The error bars represent means ± SD, *n* = 3. (**B**) Western blot analysis showing the effect of CH223191 on the AHR protein levels in MDA-MB-468 cells. Cells were treated with 10 μM of CH223191, 10 μM of Q18, or DMSO (vehicle) for 24 h. All lanes contain 60 μg of whole cell lysates. The amount of AHR was normalized by total protein stain. The normalized value of AHR from the treatment group was divided by the normalized value from DMSO treatment to calculate the relative abundance of AHR. The error bar represents mean ± SD of three independent experiments.

Results from the high-throughput gene expression screening revealed that the *ahr* and *cyp1a1* transcripts were upregulated by 2.5- and 8-fold, respectively, in MDA-MB-468 cells after treatment with 10 μM of Q18 for 24 h (unpublished data). We were able to confirm the CYP1A1 upregulation (Figure 8A); however, no upregulation of AHR at the transcript level has been observed within the 24 h treatment period of Q18 (Figure 4A). To our surprise, the AHR protein is rapidly reduced upon Q18 treatment. Even though it is unclear how CYP1A1 can be upregulated by Q18 while the AHR expression is actually suppressed, Q18 is nevertheless an effective tool to eventually help us to unveil how TNBC cells regulate the AHR protein levels without exogenous ligand treatment. AHR and its partner ARNT have been linked to autophagy. AHR levels are inversely proportional to the autophagy potential in non-small cell lung cancer cells; the activation of AHR causes less metastatic potential by downregulating autophagy, resulting in lesser EMT progression [40]. However, the activation of AHR in another cell type may trigger macroautophagy to repair cellular damage caused by external agents. For example, researchers have shown that particulate matter induced LC3/p62-mediated macroautophagy by activating AHR in keratinocytes [41]. It has been reported that autophagy may protect the liver against alcohol-induced damage, which, interestingly, is ARNT-dependent, since ARNT knockout mice were resistant to such damage [42]. Recently, we reported that AHR undergoes LC3-mediated autophagy in HeLa cells [43]. However, in contrast to what we observed here in MDA-MB-468 cells, the activation of CMA does not degrade AHR in HeLa cells. Interestingly our data support the selective degradation of AHR via CMA in TNBC cells, which adds another layer of complexity to these vital mechanisms involving autophagy.

One of the first questions we asked was whether Q18 degrades AHR via the well-established mechanism involving the proteasomal degradation of AHR after ligand binding [31]. Our data revealed that Q18 is not an AHR ligand; rather, it is an AHR antagonist. However, this Q18 effect on the AHR degradation is not mediated through its antagonistic activity since CH223191, unlike Q18, did not alter the AHR protein levels in MDA-MB-468 cells up to 24 h of treatment (Figure 8B).

In addition to the early discovery that client proteins such as RNase A and GAPDH are preferentially uptaken into lysosomes for degradation via the CMA mechanism [42], hypoxia inducible factor 1α (HIF-1α), a PAS protein-like AHR, has been reported as a CMA client protein [44,45]. Carboxyl terminus of HSC70-interacting protein (CHIP, aka STUB1), a co-chaperone E3 ligase [46,47], forms the K63-linked ubiquitinated HIF1α, which in turn triggers the degradation of HIF-1α via the LAMP2A-mediated CMA mechanism [38,48]. Interestingly, CHIP has been shown (more than a decade ago) to degrade the unliganded AHR, and this degradation can be protected in the presence of XAP2 [49]. The down-regulation of CHIP in human liver Hepa-1c1c7 cells, surprisingly, does not alter the AHR protein levels or function [50], possibly due to the cell-specific involvement of CHIP in the regulation of the AHR protein levels. This is consistent with our finding that the degradation of AHR is mediated through CMA in TNBC cells but not in non-TNBC and HeLa cells.

It is commonly known that MDA-MB-468 and MDA-MB-231 cells are ER-, PR-, and HER2-negative, whereas MCF-7 and T47D cells are ER- and PR-positive but HER2-negative. MDA-MB-361 cells, on the other hand, are ER- and HER2-positive but might express PR-B [51,52], which might explain our observation that Q18 reduces the AHR levels in MDA-MB-361 cells initially but that the levels can be quickly reverted back, since the Q18 effect is PR-B-sensitive. Q18 triggers AHR protein degradation in triple-negative but not in non-triple-negative breast cancer cells, partly because PR-B expression blocks autophagy-dependent AHR degradation. It appears that PR-B activity prevents AHR from CMA-mediated degradation—this hypothesis is consistent with the literature that progesterone increases the AHR protein levels in the uterus of rats [53] and rabbits [54]. It was reported that the activation of the PR-B signaling suppresses the AHR function in T47D cells [55]. This crosstalk should be more complicated, since PR-B can stabilize the AHR protein levels, which might contribute to any sustained action of AHR in T47D cells. Since the expression levels of LAMP2A are inversely proportional to PR-B (Figure 5I), it would be intriguing to examine whether PR-B can act as a transcriptional suppressor of the LAMP2A gene transcription or as a modulator of the LAMP2A protein half-life.

To validate whether the activation of CMA is the cause of AHR protein degradation, we subjected MDA-MB-468 cells to a condition where CMA is triggered—the treatment of 6-AN [56] or starvation [37,39,57]. The results from our proximity ligation assay prove that AHR interacts with LAMP2A in situ when CMA is activated. In all cases, with the treatment of Q18, 6-AN, or HBSS (to mimic starvation), the LAMP2A expression is enhanced, followed by an increase in the AHR-LAMP2A interaction, leading to AHR protein degradation, which can be reversed by an autophagy inhibitor chloroquine. Our data are consistent with other researchers’ observation that the treatment of Hep3B cells with digoxin, which appears to increase lysosomal degradation, increases the LAMP2A expression [45]. Increased LAMP2A expression at the lysosomal membrane is likely due to a decrease in the LAMP2A protein degradation and/or the shuttling of LAMP2A from lysosomal cytosol to membrane [58]. In addition, 6-AN and Q18 enhance the interaction between HSC70 and AHR, supporting the notion that HSC70 escorts AHR to LAMP2A for CMA. Although GA has been shown as a CMA activator after 24 h of treatment at 2 μM concentration using isolated lysosomes [56], we were not able to see any activation of autophagy at that concentration—there was no increase in the LAMP2A expression and the AHR protein levels could not be restored by chloroquine after GA treatment in MDA-MB-468 cells. We cannot rule out the possibility that the timing and dose should be adjusted if cells rather than isolated lysosomes are used for the study. Nevertheless, it is interesting that the AHR protein levels were pronouncedly suppressed after 24 h of GA treatment and neither chloroquine nor MG132 could restore the levels. It is well known that GA causes the ligand-independent degradation of AHR via the 26S proteasome and that this degradation can occur within 2 h of GA treatment [32]. It is conceivable that AHR in MDA-MB-468 cells might be degraded to a great extent shortly after GA treatment and remain at low levels for up to 24 h. Thus, exposing cells with chloroquine or MG132 at the last 8 h of the 24 h GA treatment window did not elicit any inhibitory effect on degradation. Collectively, our data strongly support the notion that TNBC cells use CMA to degrade AHR selectively and that Q18 upregulates CMA to degrade AHR. Interestingly, this Q18-dependent CMA activation is PR-B dependent and thus is not observed in non-TNBC cells, revealing potentially important crosstalk between the PR-B signaling and autophagy mechanisms in breast cancer cells.

A pentapeptide sequence KFERQ was discovered to be the signature motif of CMA client proteins [6]. The general requirement of the pentapeptide signature motif is as follows: (1) one acid residue (D, E) with two basic (K, R) and one hydrophobic (F, V, L, I) residues or (2) one acid residue (D, E) with one basic (K, R) and two hydrophobic (F, V, L, I) residues. Both scenarios should have a Q or N as the first or last residue of the pentapeptide. This signature motif is thought to involve in the HSC70-mediated interactions that brings client proteins into lysosomes for degradation. Interestingly, human AHR contains putative CMA motifs—one putative signature sequence at amino acid 558 (NEKFF) and two quasi sequences at amino acid 20 (QKTVK) and amino acid 59 (QDVIN). The deletion construct of AHR (amino acid 1-295, EGFP-CΔ553) lacking the NEKFF sequence is rather stable in MDA-MB-468 cells after transient transfection—this observation rules out the involvement of the two quasi sequences for CMA. On the other hand, when two of the five amino acids of NEKFF are mutated in the GFP fusion of AHR, GFP-AHR becomes more stable upon Q18 treatment, supporting the notion that NEKFF is a bona fide motif that is responsible for the CMA-mediated AHR degradation. This motif is remarkably similar to the human HIF-1α CMA motif (NEFKL) and, interestingly, both the human AHR and HIF-1α motif are not conserved in mouse [48]. In summary, we have provided supportive evidence to conclude that AHR is a CMA client protein which can be degraded selectively via CMA in TNBC cells. Mechanistic studies to better understand how cells utilize lysosomal degradation to regulate their AHR protein levels are warranted.

## 4. Materials and Methods

### 4.1. Cell Lines and Reagents

MDA-MB-468 cells were maintained in Advanced MEM (Gibco) supplemented with 5% FBS (Gemini Bio, West Sacramento, CA, USA), streptomycin (100 μg/mL), and penicillin (100 units/mL) (Invitrogen, Carlsbad, CA, USA). MDA-MB-231 cells were a gift from Dr. Jesika Faridi (University of the Pacific) and were grown in DMEM/F12 (1:1) (Gibco, Gaithersburg, MD, USA) with 10% FBS, streptomycin (100 μg/mL), and penicillin (100 units/mL). MCF-7 and T47D cells were obtained from ATCC. MDA-MB-361 cells were a gift from Dr. Xiaoling Li (University of the Pacific). Rat H4G1.1c3 stable cells carrying a DRE-driven GFP cDNA [59] were a gift from Dr. Michael Denison (UC Davis, Davis, CA, USA). Both MDA-MB-468 and T47D cells, which were used to generate most of the data in this manuscript, were authenticated by ATCC. Cells, if not specified, were grown in 10% FBS containing HyClone DMEM (Fisher Scientific, Waltham, MA, USA), streptomycin (100 μg/mL), and penicillin (100 units/mL). All the cells were maintained as a monolayer at 37 °C and 5% CO_2_. 3-Methylcholanthrene (3MC) was purchased from Supelco Analytical. Q18 ((4-(5-ethylpyrimidin-2-yl)piperazin-1-yl)(4-((quinazolin-4-ylamino)methyl)phenyl)methanone) was synthesized as previously described [34]. Cycloheximide (CHX) was purchased from Santa Cruz Biotechnology. MG132 was purchased from Cayman Chemical. Actinomycin D, geldanamycin (GA), chloroquine (CQ), 6-amino-nicotinamide (6-AN), CH223191, puromycin dihydrochloride, β-napthoflavone (βNF), HBSS, PMSF, and leupeptin were purchased from Sigma-Aldrich. DRE (sense: TCGAGTAGAT*CACGC*AATGGGCCCAGC and antisense: TCGAGCTGGGCCCATT*GCGTG*ATCTAC) with IRD700 conjugated at the 5′ end of both strands were purchased from Integrated DNA Technology. *Plasmids:* pcDNAHygro(+)LAMP2AS was a gift from Dr. Janice Blum (Addgene plasmid #86146). pcDNA3-PRB and pCMV-ERα were gifts from Dr. Elizabeth Wilson (Addgene plasmids #89130 and #101141). Lentiviral pLKO.1 plasmid carrying the human PR-B specific shRNA (RHS4533-EG5241, NM_000926.4) was purchased from Dharmacon (Lafayette, CO, USA). The pLKO.1 scramble shRNA plasmid was purchased from Addgene (Watertown, MA, USA). The codon humanized pGFP^2^-N2 plasmid was purchased from BioSignal Packard (Montreal, QC, Canada). The pGFP^2^-AHR plasmid was generated by cloning the full-length human AHR cDNA sequence upstream to the GFP cDNA at EcoRV site of pGFP^2^-N2. All cloned plasmids were sequenced to confirm the identity (Functional Biosciences, Madison, WI, USA). Purified plasmid was generated using the Zymopure maxiprep kit (Zymo Research, Irvine, CA, USA). *Antibodies*: Anti-AHR (SA210) polyclonal rabbit IgG was purchased from Enzo Life Sciences. Mouse monoclonal anti-LAMP2 (H4B4, sc-18822), mouse monoclonal anti-PR (B-30, sc-811), mouse monoclonal anti-GFP (B-2, sc-9996-AF790), mouse monoclonal anti-ERα (F-10, sc-8002), and mouse monoclonal anti HSC70 (B-6, sc-7298) antibodies were purchased from Santa Cruz Biotechnology (Santa Cruz, CA, USA). Rabbit anti-GAPDH polyclonal IgG G9545 was purchased from Sigma-Aldrich. Anti-β-actin monoclonal mouse IgG AM4302 was purchased from Ambion (Thermo Fisher, Waltham, MA, USA). All the secondary donkey antibodies conjugated with IRDye 800CW or 680RD were purchased from LI-COR Bioscience (Lincoln, NE, USA).

### 4.2. Western Blot Analysis

Western blot analysis was performed as described previously [60]. In brief, whole cell lysates were prepared by three cycles of freeze/thaw and then incubation on ice for 30 min in HEDG buffer (25 mM HEPES, pH 7.4, 1 mM EDTA, 1 mM DTT, 10% glycerol) containing 0.4 M KCl, 1 mM PMSF, and 2 µg/mL of leupeptin, followed by centrifugation (16,000× *g* for 10 min at 4 °C). BCA protein assay (Thermal Fisher, Waltham, MA, USA) was used to determine the protein content. Typically, 60 μg of lysates were used for Western blot analysis. The results were analyzed using a LI-COR CLx Odyssey imager. The amount of target proteins was normalized with β-actin or the total protein using the Revert total protein stain (LI-COR). Dilutions of antibodies were as follows: anti-AHR SA210, 1:4000; anti-LAMP2 H4B4, 1:200; anti-PR-B B-30, 1:200; anti-GFP B-2-AF790 conjugate, 1:200; anti-ERα F-10, 1:200; anti-HSC70 B-6, 1:200; anti-GAPDH G9545, 1:5000; anti-β-actin AM4302, 1:5000; all donkey secondary antibodies, 1:10,000.

### 4.3. Reverse Transcription-Quantitative PCR

RNA was extracted from cells using Direct-zol RNA kit (Zymo Research, Irvine, CA, USA). cDNA synthesis was performed using MMLV high-performance reverse transcriptase (Epicentre, Madison, WI, USA). RT-qPCR was performed using a Bio-Rad CFX Connect real-time PCR system as described previously [60]. All primers used were as follows: *cyp1a1* (forward: GGCCACATCCGGGACATCACAGA and reverse: TGGGGATGGTGAAGGGGACGAA), *ahr* (forward: ACATCACCTACGCCAGTCGC and reverse: TCTATGCCGCTTGGAAGGAT), *18S* (forward: CGCCCCCTCGATGCTCTTAG and reverse: CGGCGGGTCATGGGAATAAC) (Invitrogen). The fold changes of gene transcript levels between treated and vehicle control treated cells were normalized by 18S using the 2^−ΔΔ*C*q^ method [61].

### 4.4. Generation of Lentivirus-Mediated Stable Knockdown Cells

Lentivirus-mediated delivery of shRNA was performed as described previously [33]. PR-B-specific lenti shRNA or scramble shRNA pLKO.1 plasmid was transfected into AD293 cells with the viral envelope plasmid (pCMV-VSV-G) and the packaging plasmid (pCMV-dR8.2 dvpr) using Endofectin transfection reagent (Genecopoeia) to allow the packaging and amplification of the lentivirus. T47D cells were infected with the viral particles (0.5 mL) in the presence of 8 μg/mL of polybrene for 24 h. Puromycin (4 μg/mL) was used to select for stable cells exhibiting PR-B knockdown. Cells were analyzed by Western blot analysis on the seventh day after infection.

### 4.5. Transient Transfection

Transient transfection was performed using the EcoTransfect transfection reagent (Oz Biosciences, San Diego, CA, USA) following the manufacturer’s instructions. Briefly, MDA-MB-468 cells were plated at a density of 10^5^ cells per well of a 6-well plate. Four microgram of a plasmid was delivered into cells with 16 µL of EcoTransfect reagent. Cells were harvested 72 h after transfection.

### 4.6. Co-Immunoprecipitation

Protein G magnetic beads (Thermo Fisher Scientific, Waltham, MA, USA), which had been preincubated with co-immunoprecipitation antibody for 30 min at room temperature, were incubated with whole cell lysates (2 mg) overnight at 4 °C with rotation. Protein G beads were then washed with the buffer (HEDG containing 150 mM NaCl and 0.05–0.1% Tween-20) three times at 4 °C. Western blot analysis was performed to visualize the results.

### 4.7. Proximity Ligation Assay

A proximity ligation assay was performed according to the protocol of Duolink Reagents Red (Sigma-Aldrich, St. Louis, MO, USA). Briefly, cells were fixed on glass slides and permeabilized using cold methanol and then incubated with corresponding rabbit and mouse antibodies for 1 h at 37 °C. Anti-rabbit PLUS and anti-mouse MINUS probes were added and incubated for another hour at 37 °C. Ligation (1:40 dilution, 30 min at 37 °C) and polymerization (1:80 dilution, 100 min at 37 °C) were then performed successively. Slides were then mounted before imaging with DAPI for 2 min at room temperature. Fluorescence images were acquired using a Keyence BZ-X700 fluorescence microscope.

### 4.8. Site-Directed Mutagenesis

pGFP^2^-AHR E559A/F561A and E559A/F561L mutants were created using the QuikChange lightning site-directed mutagenesis kit according to the manufacturer’s protocol (Stratagene). Mutant strand was amplified by PCR reaction containing 10 ng of template DNA (pGFP^2^-AHR) and 125 ng of each primer (Forward: 5′-GACATCAGACACATGCAGAATGCAAAAGCTTTCAGAAATGA-TTTTTCTGGTGAGG-3′, Reverse: 5′-CCTCACCAGAAAAATCATTTCTGAAAGCTTTTG-CATTCTGCATGTGTCTGATGTC-3′). XL10-Gold ultracompetent cells were used for transformation. Originally, E559A/F561A was designed, but DNA sequencing results confirmed that we had both E559A/F561A and E559A/F561L mutants.

### 4.9. Gel Shift Assay

Pichia expressed human AHR and ARNT were used to form the ligand-dependent AHR gel shift complex, as described previously [35], with modifications. In brief, 6His-AHR and 6His-ARNT expressed in 9 mL and 2 mL of Pichia culture, respectively, were enriched by metal affinity purification into 0.75 mL of HEDG except with 50% glycerol. 6His-human p23 from 600 mL of bacterial culture was likewise enriched into 1.5 mL of PBS and was used to promote the formation of the AHR gel shift complex [36]. AHR gel shift complex was formed by adding the AHR ligand βNF (10 μM) into HEDG containing AHR (2 μL), ARNT (0.5 μL), and p23 (1 μL), followed by addition of dIdC (3 μg) and DRE-IRD700 (0.5 pmol). A LI-COR CLx imager was used to visualize the results when electrophoresis was complete.

### 4.10. Statistical Analysis

All the data are reported as means ± SD and analyzed using Prism GraphPad version 8. Statistical analysis was determined by one-way or two-way ANOVA with multiple comparisons, or Student’s *t* test. The number of asterisk indicates the *p* value of statistical significance as follows: *p* > 0.05 (ns, not significant), *p* < 0.05 (*), *p* < 0.01 (**), *p* < 0.001 (***), and *p* < 0.0001 (****).

## 5. Conclusions

Similar to 6-AN and starvation, Q18 promotes CMA in MDA-MB-468 cells. The activation of CMA in these cells triggers the degradation of the AHR protein selectively, since human AHR contains a CMA-like motif at amino acid 558-562 (NEKFF), which is essential for this degradation. This degradation is mediated through LAMP2A and appears to occur in breast cancer cells when PR-B is lacking.

## Abbreviations

AHR	aryl hydrocarbon receptor
ARNT	aryl hydrocarbon receptor nuclear translocator
HSP90	heat shock protein 90
HSC70	heat shock cognate protein 70
DRE	dioxin response element
CMA	chaperone-mediated autophagy
3MC	3-methylcholanthrene
βNF	β-naphthoflavone
HIF-1α	hypoxia inducible factor 1 alpha
CHIP	carboxy terminus of HSC70-interacting protein
LAMP2A	lysosomal associated membrane protein 2A
PR-B	progesterone receptor B
ERα	estrogen receptor alpha
Q18	(4-(5-ethylpyrimidin-2-yl)piperazin-1-yl)(4-((quinazolin-4 ylamino)methyl)phenyl)methanone
CHX	cycloheximide
CQ	chloroquine
6-AN	6-amino-nicotinamide
GA	geldanamycin
CYP	cytochrome P450
Act D	actinomycin D

## References

[1] Mizushima N., Levine B., Cuervo A.M., Klionsky D.J. (2008). Autophagy fights disease through cellular self-digestion. Nature.

[2] Majeski A.E., Dice J.F. (2004). Mechanisms of chaperone-mediated autophagy. Int. J. Biochem. Cell Biol..

[3] Kaushik S., Cuervo A.M. (2018). The coming of age of chaperone-mediated autophagy. Nat. Rev. Mol. Cell Biol..

[4] Cuervo A.M., Dice J.F. (2000). Unique properties of lamp2a compared to other lamp2 isoforms. J. Cell Sci..

[5] Bandyopadhyay U., Kaushik S., Varticovski L., Cuervo A.M. (2008). The chaperone-mediated autophagy receptor organizes in dynamic protein complexes at the lysosomal membrane. Mol. Cell. Biol..

[6] Chiang H.-L., Dice J.F. (1988). Peptide sequences that target proteins for enhanced degradation during serum withdrawal. J. Biol. Chem..

[7] Burbach K.M., Poland A., Bradfield C.A. (1992). Cloning of the Ah-receptor cDNA reveals a distinctive ligand-activated transcription factor. Proc. Natl. Acad. Sci. USA.

[8] Schmidt J.V., Su G.H., Reddy J.K., Simon M.C., Bradfield C.A. (1996). Characterization of a murine Ahr null allele: Involvement of the Ah receptor in hepatic growth and development. Proc. Natl. Acad. Sci. USA.

[9] Gonzalez F.J., Fernandez-Salguero P. (1998). The aryl hydrocarbon receptor: Studies using the AHR-null mice. Drug Metab. Dispos..

[10] Nguyen N.T., Kimura A., Nakahama T., Chinen I., Masuda K., Nohara K., Fujii-Kuriyama Y., Kishimoto T. (2010). Aryl hydrocarbon receptor negatively regulates dendritic cell immunogenicity via a kynurenine-dependent mechanism. Proc. Natl. Acad. Sci. USA.

[11] Mezrich J.D., Fechner J.H., Zhang X., Johnson B.P., Burlingham W.J., Bradfield C.A. (2010). An interaction between kynurenine and the aryl hydrocarbon receptor can generate regulatory T cells. J. Immunol..

[12] Boitano A.E., Wang J., Romeo R., Bouchez L.C., Parker A.E., Sutton S.E., Walker J.R., Flaveny C.A., Perdew G.H., Denison M.S. (2010). Aryl hydrocarbon receptor antagonists promote the expansion of human hematopoietic stem cells. Science.

[13] Schlezinger J.J., Liu D., Farago M., Seldin D.C., Belguise K., Sonenshein G.E., Sherr D.H. (2006). A role for the aryl hydrocarbon receptor in mammary gland tumorigenesis. Biol. Chem..

[14] Kolluri S.K., Jin U.H., Safe S. (2017). Role of the aryl hydrocarbon receptor in carcinogenesis and potential as an anti-cancer drug target. Arch. Toxicol..

[15] Silginer M., Burghardt I., Gramatzki D., Bunse L., Leske H., Rushing E.J., Hao N., Platten M., Weller M., Roth P. (2016). The aryl hydrocarbon receptor links integrin signaling to the TGF-beta pathway. Oncogene.

[16] Seymour E.M., Bennink M.R., Bolling S.F. (2013). Diet-relevant phytochemical intake affects the cardiac AhR and nrf2 transcriptome and reduces heart failure in hypertensive rats. J. Nutl. Biochem..

[17] Wang Q., Fan Y., Kurita H., Jiang M., Koch S., Rao M.B., Rubinstein J., Puga A. (2017). Aryl hydrocarbon receptor ablation in cardiomyocytes protects male mice from heart dysfunction induced by NKX2.5 haploinsufficiency. Toxicol. Sci..

[18] Wong P.S., Vogel C.F., Kokosinski K., Matsumura F. (2010). Aryl hydrocarbon receptor activation in NCI-H441 cells and C57BL/6 mice: Possible mechanisms for lung dysfunction. Am. J. Respir. Cell Mol. Biol..

[19] Lee S.M., Park H.Y., Suh Y.S., Yoon E.H., Kim J., Jang W.H., Lee W.S., Park S.G., Choi I.W., Choi I. (2017). Inhibition of acute lethal pulmonary inflammation by the IDO-AhR pathway. Proc. Natl. Acad. Sci. USA.

[20] Zhang L., Hatzakis E., Nichols R.G., Hao R., Correll J., Smith P.B., Chiaro C.R., Perdew G.H., Patterson A.D. (2015). Metabolomics reveals that aryl hydrocarbon receptor activation by environmental chemicals induces systemic metabolic dysfunction in mice. Environ. Sci. Technol..

[21] Belton K.R., Tian Y., Zhang L., Anitha M., Smith P.B., Perdew G.H., Patterson A.D. (2018). Metabolomics reveals aryl hydrocarbon receptor activation induces liver and mammary gland metabolic dysfunction in lactating mice. J. Proteome Res..

[22] Choudhary M., Kazmin D., Hu P., Thomas R.S., McDonnell D.P., Malek G. (2015). Aryl hydrocarbon receptor knock-out exacerbates choroidal neovascularization via multiple pathogenic pathways. J. Pathol..

[23] Gutierrez M.A., Davis S.S., Rosko A., Nguyen S.M., Mitchell K.P., Mateen S., Neves J., Garcia T.Y., Mooney S., Perdew G.H. (2016). A novel AhR ligand, 2AI, protects the retina from environmental stress. Sci. Rep..

[24] Carver L.A., Bradfield C.A. (1997). Ligand-dependent interaction of the aryl hydrocarbon receptor with a novel immunophilin homolog in vivo. J. Biol. Chem..

[25] Ma Q., Whitlock J.P. (1997). A novel cytoplasmic protein that interacts with the Ah receptor, contains tetratricopeptide repeat motifs, and augments the transcriptional response to 2,3,7,8-tetrachlorodibenzo-*p*-dioxin. J. Biol. Chem..

[26] Petrulis J.R., Kusnadi A., Ramadoss P., Hollingshead B., Perdew G.H. (2003). The hsp90 co-chaperone XAP2 alters importin beta recognition of the bipartite nuclear localization signal of the Ah receptor and represses transcriptional activity. J. Biol. Chem..

[27] Kazlauskas A., Sundstrom S., Poellinger L., Pongratz I. (2001). The hsp90 chaperone complex regulates intracellular localization of the dioxin receptor. Mol. Cell. Biol..

[28] Kazlauskas A., Poellinger L., Pongratz I. (1999). Evidence that the co-chaperone p23 regulates ligand responsiveness of the dioxin (Aryl hydrocarbon) receptor. J. Biol. Chem..

[29] Nebert D.W., Puga A., Vasiliou V. (1993). Role of the Ah receptor and the dioxin-inducible [Ah] gene battery in toxicity, cancer, and signal transduction. Ann. N. Y. Acad. Sci..

[30] Vogel C.F., Sciullo E., Li W., Wong P., Lazennec G., Matsumura F. (2007). RelB, a new partner of aryl hydrocarbon receptor-mediated transcription. Mol. Endocrinol..

[31] Davarinos N.A., Pollenz R.S. (1999). Aryl hydrocarbon receptor imported into the nucleus following ligand binding is rapidly degraded via the cytosplasmic proteasome following nuclear export. J. Biol. Chem..

[32] Pollenz R.S., Buggy C. (2006). Ligand-dependent and -independent degradation of the human aryl hydrocarbon receptor (hAHR) in cell culture models. Chem. Biol. Interact..

[33] Nguyen P.M., Wang D., Wang Y., Li Y., Uchizono J.A., Chan W.K. (2012). p23 co-chaperone protects the aryl hydrocarbon receptor from degradation in mouse and human cell lines. Biochem. Pharmacol..

[34] Shallal H.M., Russu W.A. (2011). Discovery, synthesis, and investigation of the antitumor activity of novel piperazinylpyrimidine derivatives. Eur. J. Med. Chem..

[35] Zheng Y., Xie J., Huang X., Dong J., Park M.S., Chan W.K. (2016). Binding studies using Pichia pastoris expressed human aryl hydrocarbon receptor and aryl hydrocarbon receptor nuclear translocator proteins. Protein Expr. Purif..

[36] Shetty P.V., Bhagwat B.Y., Chan W.K. (2003). p23 enhances the formation of the aryl hydrocarbon receptor-DNA complex. Biochem. Pharmacol..

[37] Massey A.C., Kaushik S., Sovak G., Kiffin R., Cuervo A.M. (2006). Consequences of the selective blockage of chaperone-mediated autophagy. Proc. Natl. Acad. Sci. USA.

[38] Ferreira J.V., Soares A.R., Ramalho J.S., Pereira P., Girao H. (2015). K63 linked ubiquitin chain formation is a signal for HIF1A degradation by chaperone-mediated autophagy. Sci. Rep..

[39] Cuervo A.M., Knecht E., Terlecky S.R., Dice J.F. (1995). Activation of a selective pathway of lysosomal proteolysis in rat liver by prolonged starvation. Am. J. Physiol..

[40] Tsai C.H., Li C.H., Cheng Y.W., Lee C.C., Liao P.L., Lin C.H., Huang S.H., Kang J.J. (2017). The inhibition of lung cancer cell migration by AhR-regulated autophagy. Sci. Rep..

[41] Jang H.S., Lee J.E., Myung C.H., Park J.I., Jo C.S., Hwang J.S. (2019). Particulate matter-induced aryl hydrocarbon receptor regulates autophagy in keratinocytes. Biomol. Ther..

[42] Ni H.M., Bhakta A., Wang S., Li Z., Manley S., Huang H., Copple B., Ding W.X. (2014). Role of hypoxia inducing factor-1beta in alcohol-induced autophagy, steatosis and liver injury in mice. PLoS ONE.

[43] Yang Y., Chan W.K. (2020). Selective Autophagy Maintains the Aryl Hydrocarbon Receptor Levels in HeLa Cells: A Mechanism That Is Dependent on the p23 Co-Chaperone. Int. J. Mol. Sci..

[44] Bento C.F., Fernandes R., Ramalho J., Marques C., Shang F., Taylor A., Pereira P. (2010). The chaperone-dependent ubiquitin ligase CHIP targets HIF-1alpha for degradation in the presence of methylglyoxal. PLoS ONE.

[45] Hubbi M.E., Hu H., Ahmed I., Levchenko A., Semenza G.L. (2013). Chaperone-mediated autophagy targets hypoxia-inducible factor-1alpha (HIF-1alpha) for lysosomal degradation. J. Biol. Chem..

[46] Cao Z., Li G., Shao Q., Yang G., Zheng L., Zhang T., Zhao Y. (2016). CHIP: A new modulator of human malignant disorders. Oncotarget.

[47] Ranek M.J., Stachowski M.J., Kirk J.A., Willis M.S. (2018). The role of heat shock proteins and co-chaperones in heart failure. Phil. Trans. R. Soc. B.

[48] Ferreira J.V., Fofo H., Bejarano E., Bento C.F., Ramalho J.S., Girao H., Pereira P. (2013). STUB1/CHIP is required for HIF1A degradation by chaperone-mediated autophagy. Autophagy.

[49] Lees M.J., Peet D.J., Whitelaw M.L. (2003). Defining the role for XAP2 in stabilization of the dioxin receptor. J. Biol. Chem..

[50] Morales J.L., Perdew G.H. (2007). Carboxyl terminus of hsc70-interacting protein (CHIP) can remodel mature aryl hydrocarbon receptor (AhR) complexes and mediate ubiquitination of both the AhR and the 90 kDa heat-shock protein (hsp90) in vitro. Biochemistry.

[51] Dai X., Cheng H., Bai Z., Li J. (2017). Breast Cancer Cell Line Classification and its relevance with breast tumor subtyping. J. Cancer.

[52] Mota A.L., Evangelista A.F., Macedo T., Oliveira R., Scapulatempo-Neto C., Vieira R.A., Marques M.M.C. (2017). Molecular characterization of breast cancer cell lines by clinical immunohistochemical markers. Oncol. Lett..

[53] Rataj F., Moller F.J., Jahne M., Honscheid P., Zierau O., Vollmer G., Kretzschmar G. (2015). Progesterone, as well as 17beta-estradiol, is important for regulating AHR battery homoeostasis in the rat uterus. Arch. Toxicol..

[54] Hasan A., Fischer B. (2001). Hormonal control of aryl hydrocarbon receptor (AhR) expression in the preimplantation rabbit uterus. Anat. Embryol..

[55] Kuil C.W., Brouwer A., Van der Saag P.T., Van der Burg B. (1998). Interference between progesterone and dioxin signal transduction pathways. Different mechanisms are involved in repression by the progesterone receptor A and B isoforms. J. Biol. Chem..

[56] Finn P.F., Mesires N.T., Vine M., Dice J.F. (2005). Effects of small molecules on chaperone-mediated autophagy. Autophagy.

[57] Slot L.A., Lauridsen A.M., Hendil K.B. (1986). Intracellular protein degradation in serum-deprived human fibroblasts. Biochem. J..

[58] Cuervo A.M., Dice J.F. (2000). Regulation of lamp2a levels in the lysosomal membrane. Traffic.

[59] Peters A.K., Leonards P.E., Zhao B., Bergman A., Denison M.S., Van den Berg M. (2006). Determination of in vitro relative potency (REP) values for mono-ortho polychlorinated biphenyls after purification with active charcoal. Toxicol. Lett..

[60] Chen J., Yakkundi P., Chan W.K. (2019). Down-regulation of p23 in normal lung epithelial cells reduces toxicities from exposure to benzo[a]pyrene and cigarette smoke condensate via an aryl hydrocarbon receptor-dependent mechanism. Toxicol. Sci..

[61] Livak K.J., Schmittgen T.D. (2001). Analysis of relative gene expression data using real-time quantitative PCR and the 2^−^^ΔΔCT^ Method. Methods.

